# A Case Report and 31-Case Study: Does Takotsubo Cardiomyopathy in Myasthenia Gravis Patients Have a High Mortality Rate?

**DOI:** 10.7759/cureus.28625

**Published:** 2022-08-31

**Authors:** Scott Gayfield, Joshua Busken, Sarmed Mansur

**Affiliations:** 1 Internal Medicine, The University of Toledo, Toledo, USA; 2 Hospital Medicine, The University of Toledo, Toledo, USA

**Keywords:** broken-heart syndrome, myasthenia gravis, myasthenic crisis, takotsubo cardiomyopathy, stress-induced cardiomyopathy

## Abstract

Myasthenia gravis is an autoimmune disorder in which antibodies are formed against post-synaptic nicotinic acetylcholine receptors that lead to impeded muscle contraction and commonly affects the oculomotor muscles. Takotsubo cardiomyopathy (TTC) is a dilated cardiomyopathy that can mimic a myocardial infarction and causes reversible systolic dysfunction. This is a case of a 66-year-old Caucasian male with a known history of ocular myasthenia gravis that presented to the emergency room with worsening dyspnea secondary to a myasthenic crisis. One day, following admission, his shortness of breath failed to improve and was found to meet the diagnostic criteria for takotsubo cardiomyopathy. A brief review of 31 previous cases summarizes the current case reports, patterns, and mortality associated with the myasthenic crisis associated with TTC.

## Introduction

Myasthenia gravis (MG) is a rare autoimmune neuromuscular disorder that classically is caused by antibodies targeting post-synaptic nicotinic acetylcholine receptors (AChR). In a minority of cases, antibodies are targeted at muscle-specific receptor tyrosine kinase (MuSK) proteins [1]. Myasthenia gravis presents with increasing weakness of skeletal muscle tissue with repeated contractions, which is often first recognized by its effects on the ocular muscles. Takotsubo cardiomyopathy (TTC) is a syndrome characterized by transient systolic dysfunction, most commonly involving the left ventricle. This syndrome is commonly preceded by prolonged physical or emotional stress and is most prevalent in post-menopausal women [2]. Clinically, TTC patients often present with chest pain, dyspnea, and in severe cases, cardiogenic shock [3,4]. Up to 96% of patients with TTC will make a full recovery within several months of presentation [5].

We present a 66-year-old Caucasian male with a history of ocular myasthenia gravis who presented in a myasthenic crisis that was later diagnosed to have features suggestive of takotsubo cardiomyopathy.

## Case presentation

The patient is a 66-year-old Caucasian male who presented to the emergency department for a five-day history of dyspnea and worsening orthopnea. He had a known history of chronic obstructive pulmonary disease (COPD) diagnosed three years prior as well as a recent diagnosis of ocular MG three months before admission with positive antibodies against the post-synaptic acetylcholine receptor (AChR) of 17.0 nmol/L (reference range: 0-0.5 nmol/L). He had been taking 60 mg prednisone daily for this diagnosis and reportedly missed an appointment with a specialist prior to admission who was planning to transition him to chronic treatment. Initial physical exam was significant for heart rate 129 beats/minute, respiratory rate 22 breaths/minute, blood pressure 154/84, bilateral ptosis, 5/5 motor strength globally, and diminished airflow in bilateral lungs with crackles in the left base. There was no evidence of jugular venous distension or peripheral edema. Labs performed in the emergency department were significant for brain natriuretic peptide (BNP) 111 pg/mL (reference range: <100 pg/mL), WBC 16.8 x 109/L (reference range: 4.0-11.0 x 109/L), alanine transaminase (ALT) 126 U/L (reference range: 0-40 U/L), aspartate aminotransferase (AST) 67 U/L (reference range: 0-41 U/L), partial pressure of oxygen (PO2) 61 mmHg (reference range: 80-100 mmHg), partial pressure of carbon dioxide (PCO2) 49 (reference range: 35-45 mmHg), bicarbonate (HCO3) 28 mmol/L (reference range: 22-26 mmol/L), troponin I 0.04 ng/mL (reference range: 0.00-0.04 ng/mL). Chest X-ray revealed prominent pulmonary vasculature suggestive of possible early congestive heart failure. The computed tomography angiography (CTA) chest showed no evidence of a pulmonary embolism or thymoma. The patient was started on breathing treatments and resumed on his home prednisone dose (60 mg once daily) for treatment of myasthenic crisis and possible COPD exacerbation.

**Figure 1:**
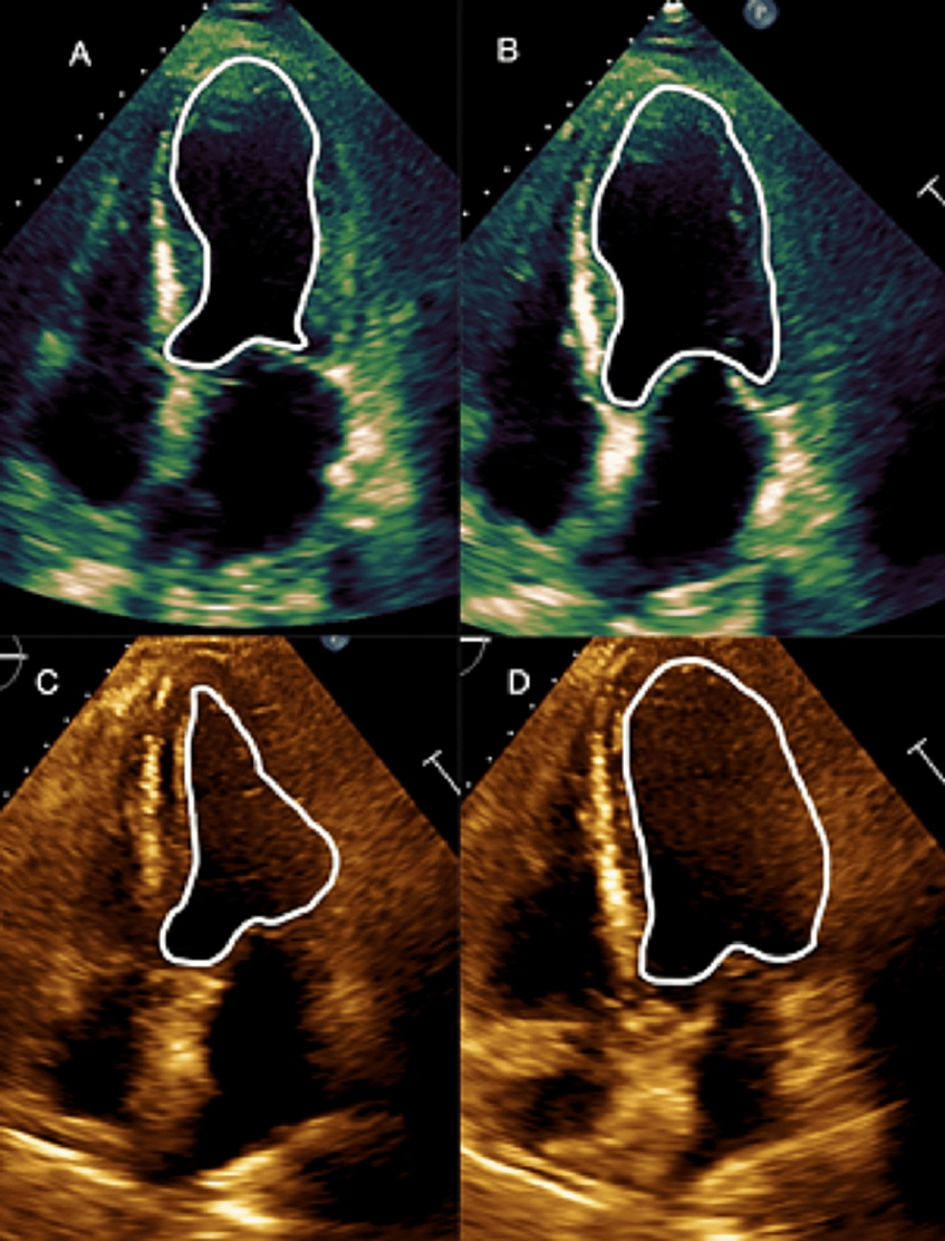
Echocardiography findings depicting the left ventricle A: Pre-treatment systole displaying apical and mid-ventricular hypokinesis, B: Pre-treatment diastole, C: Post-treatment systole displaying appropriate left ventricle (LV) contraction, D: Post-treatment diastole

**Figure 2:**
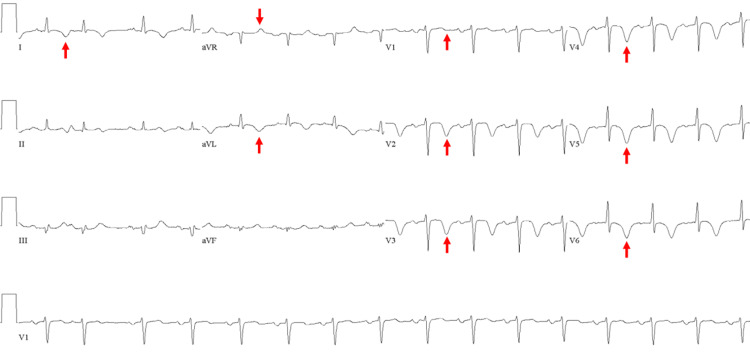
Approximately 24 hours after admission, the patient was found to have an elevated troponin despite an initially negative troponin and ECG. The repeat ECG, shown here, displays sinus rhythm with T wave inversion (red arrows) in the anterolateral leads (leads I, aVR, aVL, V1-V6) and prolonged QTc interval.

One day after admission, the patient continued to have worsening respiratory muscle weakness and shortness of breath that was refractory to breathing treatments as well as new-onset bilateral lower extremity edema. Transthoracic echocardiogram revealed left ventricular systolic dysfunction with reduced ejection fraction (25% to 30%), left ventricular wall motion abnormalities including apical and middle hypokinesis with basal normokinesis consistent with takotsubo cardiomyopathy, and moderately elevated right-sided pressures (Figure 1). Repeat troponin I was elevated at 1.69 ng/mL with the electrocardiogram (ECG) showing T wave inversions in the anterior leads with prolonged QTc interval (Figure 2). He denied chest pain and was treated with aspirin and heparin drip. Due to these findings, the patient was taken urgently to the cardiac catheterization lab for left heart catheterization (LHC). The LHC revealed mild to moderate three-vessel coronary artery disease and elevated right-sided pressures without any evidence of acute arterial pathology. With a combination of echocardiography and the absence of obstructive coronary pathology, the patient was diagnosed with takotsubo cardiomyopathy. The patient was transferred to the ICU for management of his worsening myasthenic crisis and required bilevel positive airway pressure (BiPAP) to maintain his respiratory status. The patient remained stable on BiPAP and did not require intubation. He was started on medical management for systolic heart failure and furosemide for his medical regimen. The combination of BiPAP and medical optimization of the systolic heart failure significantly improved his dyspnea. He was medically optimized for systolic heart failure with lisinopril, carvedilol, aspirin, statin, and furosemide.

The patient was discharged hemodynamically stable on room air. He was discharged on prednisone 60 mg once daily for his myasthenia gravis with a follow-up appointment with a specialist within one week of discharge. A follow-up appointment with cardiology was also scheduled for medical management of his systolic heart failure. The patient was discharged with a LifeVest™ (ZOLL Medical Corp., Pittsburgh, PA, USA) as well as a short course of furosemide with a scheduled lab follow-up four days after discharge for the monitoring of his electrolytes.

## Discussion

Myasthenia gravis is an autoimmune-mediated pathologic process in which antibiotics are directed against the post-synaptic membranes of the neuromuscular junction including most commonly the post-synaptic nicotinic acetylcholine receptor (AChR) and muscle-specific tyrosine kinase (MuSK) antibodies. These antibodies are present in approximately 90% of patients and those without these antibodies are deemed seronegative [6]. Often, the oculomotor muscles are affected first and present with ptosis and dystopia. However, the symptoms can also become more generalized with time. Myasthenia gravis can therefore be categorized as either ocular myasthenia or generalized myasthenia depending on whether the disease is limited to the ocular muscles or has become widespread [7]. Individuals with seropositive MG are also at elevated risk of secondary complications including, most notably, a neoplasm of the thymus. Patients with MG are often treated with a combination of acetylcholinesterase inhibitors, immunosuppression, and thymectomy [1,7,8]. Dangerously, patients with underlying MG are at risk of developing acute myasthenic crisis, which is a life-threatening exacerbation of myasthenic weakness that can lead to respiratory depression and possibly even death. In patients with known MG, the annual prevalence of myasthenic crisis is approximately 2% to 3% [9] and a lifetime prevalence of myasthenic crisis is predicted to be 10% to 20% [10]. In a patient with a known history of MG that presents with sudden onset weakness and decreased respiratory effort, differential diagnoses should include both myasthenic crisis and cholinergic crisis. Our patient was not on any anticholinesterase medications and therefore ruled out a cholinergic crisis.

General treatment of myasthenic crisis includes ICU management, ventilatory support, immunotherapy, and rapid therapies [11]. Respiratory support comes in the form of either noninvasive ventilation (BiPAP) or endotracheal intubation depending on the status of the patient’s respiratory muscle strength either via clinical and/or objective parameters such as their vital capacity (VC) and maximum inspiratory pressure (MIP). Immunotherapy with corticosteroids such as prednisone 60 mg or 80 mg once daily is often used for the treatment of myasthenic crisis. However, initiation should be cautioned by the possibility of intermittent worsening of respiratory function that will improve with time [11]. Intravenous immunoglobulin (IVIG) and plasmapheresis are also options for myasthenic crisis and have been shown to be beneficial primarily in severe myasthenic crisis [11]. Our patient did not receive IVIG or plasmapheresis due to clinical judgment by the ICU team determining that his crisis was not severe enough to warrant these therapies.

Takotsubo cardiomyopathy (TTC), also known as stress-induced cardiomyopathy and broken heart syndrome, is characterized primarily by transient regional systolic dysfunction, usually involving the left ventricle. While symptoms of chest pain in addition to ECG and cardiac enzymes can mimic myocardial infarction, patients with TTC undergo coronary catheterization that is negative for any obstructive coronary artery disease or acute plaque rupture. Additionally, an echocardiogram will display transient wall abnormalities, most often hypokinesis of the left ventricular apical and middle-ventricular segments. The TTC is diagnosed by meeting all conditions of the Mayo Clinic diagnostic criteria [12]. While the exact mechanism of TTC is unknown, it is thought to be primarily due to catecholamine surge leading to microvascular spasm and disruption of myocytes through calcium overload and interference with signal transduction pathways [13]. This can mimic acute myocardial infarction including elevated cardiac enzymes and ECG changes [2]. Interestingly, the percentage of TTC cases associated with neuropsychiatric disorders is more than double the rate that is seen in acute coronary syndrome [2].

Our patient presented with respiratory muscle weakness and associated shortness of breath in the setting of seropositive ocular MG diagnosed three months prior, for which he was taking prednisone 60 mg once daily. Upon presentation, his ocular symptoms were similar to his baseline and he did not have limb weakness. Initially, his primary differential diagnoses were a myasthenic crisis, COPD exacerbation, and heart failure. While the BNP was elevated at 111 pg/mL, the patient was otherwise clinically euvolemic without evidence of jugular venous distension or lower extremity edema. In the setting of a clinically euvolemic patient, our team had a low suspicion for the diagnosis of heart failure despite the elevated BNP. It was difficult to initially discern between myasthenic crisis and COPD exacerbation due to the acute nature of the presentation. While no objective measurements of the respiratory strength were measured, such as VC or MIP, the worsening of the dyspnea requiring continuous BiPAP despite steroid and bronchodilator therapy led our team to approach myasthenic crisis as the primary diagnosis. Additionally, our team identified the importance of having a low threshold of suspicion for a myasthenic crisis in a patient with a known history of MG, as it can be deadly without early intervention.

It was not until one day after admission that the patient developed findings of bilateral lower extremity edema which warranted an echocardiogram. The echocardiogram showed classic features of TTC with apical hypokinesis and basal normokinesis (as seen above in Figure 1) as well as a greater than 50% reduction in his inspiratory effort that was thought to possibly represent the muscle weakness seen and myasthenic crisis. These echocardiogram findings in the setting of his ECG findings, hypertroponinemia, and lack of acute obstructive coronary disease on LHC fulfilled three of the four Mayo Clinic diagnostic criteria for TTC. The final criterion requires ruling out the presence of pheochromocytoma and myocarditis [12]. While no inflammatory markers were drawn for myocarditis, the wall motion abnormalities of myocarditis tend to be more global in nature compared to the regional nature of TTC [14]. If the wall motion abnormalities of myocarditis are regional, they less commonly represent the specific LV apical and mid-ventricular dysfunction seen in our patient [14]. Metanephrine levels were not drawn to rule out pheochromocytoma. While our patient did have hypertension and tachycardia upon presentation, a history of headaches, sweating, and palpitations is present in up to 95% of patients with pheochromocytoma [15], none of which our patient had. This made pheochromocytoma an unlikely diagnosis and it was not pursued further with metanephrine testing.

A literature review of TTC associated with myasthenic crisis revealed 19 cases on PubMed and 13 additional cases found on Google Scholar and Elton B Stephens Co. (EBSCO) host. One case was unable to be fully accessed, leaving 31 total cases for our review and 32 total cases including our own. As seen in Table 1, the age range of reported cases is 34 to 85 years with a median of 69.5 years. Females seem to be disproportionately affected by this pathology (21/32, 65.6%). A review by Templin et al. displayed that TTC is not necessarily associated with a myasthenic crisis but is similarly predominant in women, citing 89.8% [2]. Per our review, dyspnea is the most common presenting feature of TTC (29/32, 90.6%). Interestingly, our patient was one of only two total cases (6.3%) reported in which the patient presented with orthopnea. Troponin was reportedly elevated in 12 of the cases (37.5%), however, many cases did not comment on their troponin results, and it was undeterminable if the test was not mentioned or was negative. This is believed to be an underestimate of the prevalence of elevated troponin in these cases, as this is found in approximately 87% of TTC cases [2] and we believe that it would not be reduced in prevalence due to a myasthenic crisis being associated with TTC in our review. However, per the Mayo Clinic diagnostic criteria of TTC, troponin elevation is not needed to fulfill the criteria if there are ECG findings such as ST-segment elevation or T wave inversions [12]. Of the patients previously diagnosed with MG prior to the myasthenic crisis and associated TTC, only three patients (9.4%), including our own, had a history of ocular MG. All other patients had a known history of generalized MG. While current mortality for myasthenic crisis is estimated to be 4% [10] and TTC is approximately 2.4% [16] our analysis shows that TTC associated with the myasthenic crisis has a mortality of 15.6% of patients. It has been previously shown that TTC associated with neurological causes has higher mortality [17]. This suggests that while both disease processes are dangerous on their own, occurring together leads to an unfortunate synergistic effect that significantly increases mortality. It should be noted that this review is primarily limited by the inability to screen all reviewed papers for the fulfillment of all four Mayo Clinic diagnostic criteria of TTC.

**Table 1:** Reported cases of myasthenic crisis-associated takotsubo cardiomyopathy MG: Myasthenia gravis, TTC: Takotsubo cardiomyopathy, ECG: Electrocardiogram, AChRAb: Acetylcholine receptor antibody, M: Male, F: Female, N/A: Not applicable, RVR: Rapid ventricular response

Age	Gender	Known history of MG	Type of MG (ocular vs. generalized)	Presenting symptoms and signs of TTC	ECG changes	Troponin elevation	AChRAb status	Thymoma	Outcome	Reference
66	M	Yes	Ocular	Dyspnea	T wave inversion, QTc prolongation	Yes	Positive	No	Recovered	Gayfield et al.
50	F	No	N/A	Hypotension	ST-segment depression, T wave inversion	-	-	No	Recovered	[18]
34	F	No	N/A	Dyspnea	Non-specific T wave changes	Yes	Positive	No	Recovered	[19]
83	F	No	N/A	Dyspnea hypotension	T wave inversion	-	Positive	No	Recovered	[20]
77	M	No	N/A	Dyspnea	ST-segment elevation	Yes	Positive	No	Recovered	[21]
69	F	Yes	Generalized	Chest pain dyspnea	ST-segment elevation	Yes	Positive	No	Recovered	[22]
60	F	Yes	Generalized	Chest pain dyspnea	ST-segment elevation	Yes	Positive	No	Recovered	[23]
40	F	Yes	Generalized	Dyspnea hypotension	T wave inversion	Yes	-	Yes	Died	[24]
69	F	Yes	Generalized	Dyspnea chest pain	ST-segment elevation	Yes	-	Unknown	Recovered	[25]
49	M	Yes	Generalized	Dyspnea	T wave inversion	Yes	-	Yes	Recovered	[26]
82	F	Yes	Generalized	Dyspnea chest pain	T wave inversion	Yes	-	Unknown	Recovered	[27]
70	M	No	N/A	Chest pain dyspnea	ST-segment elevation	Yes	Positive	No	Recovered	[28]
63	F	Yes	Ocular	Dyspnea	T wave inversion, QTc prolongation	-	Positive	No	Recovered	[29]
64	M	Yes	Generalized	Dyspnea chest pain	ST-segment elevation	Yes	-	No	Recovered	[30]
75	F	Yes	Generalized	Dyspnea	ST-segment elevation	Yes	-	No	Recovered	[31]
83	F	No	N/A	Dyspnea	ST-segment elevation, T wave inversion	-	Positive	Unknown	Recovered	[32]
52	F	No	N/A	Dyspnea	ST-segment elevation	-	Positive	Yes	Recovered	[33]
42	F	Yes	Ocular	Dyspnea	-	-	Positive	Yes	Recovered	[34]
75	F	Yes	Generalized	Dyspnea	T wave inversion, QTc prolongation	Yes	-	Unknown	Recovered	[35]
64	F	Yes	Generalized	Chest pain dyspnea	None	Yes	-	Unknown	Recovered	[36]
85	M	No	N/A	Dyspnea	ST-segment elevation	Yes	Negative	Unknown	Died	[37]
76	F	No	N/A	Coughing apnea	Insignificant	Yes	Positive	Yes	Died	[38]
59	F	Yes	Generalized	Orthopnea	ST-segment elevation	Yes	Positive	Unknown	Recovered	[39]
72	M	Yes	Generalized	Dysphagia dyspnea	ST-segment elevation	Yes	-	Unknown	Died	[40]
81	M	No	N/A	Dyspnea	ST-segment elevation, T wave inversion	Yes	Positive	Unknown	Died	[41]
67	F	Yes	Generalized	Dyspnea	T wave inversion, QTc prolongation	Yes	-	Unknown	Recovered	[42]
70	F	Yes	Generalized	Dyspnea	Insignificant	Yes	-	Yes	Recovered	[43]
77	M	Yes	Generalized	Dyspnea	Non-ST elevation	Yes	-	Unknown	Recovered	[44]
70	F	Yes	Generalized	Dyspnea	Atrial fibrillation with RVR	-	Negative	Unknown	Recovered	[45]
39	F	No	N/A	Dyspnea	ST-segment elevation	Yes	Positive	Unknown	Recovered	[46]
81	M	Yes	Generalized	Dyspnea	Ventricular tachycardia	Yes	-	Unknown	Recovered	[47]
74	M	Yes	Generalized	Dyspnea	-	Yes	-	Yes	Recovered	[48]

## Conclusions

Here, we report the first known case of a patient with previously diagnosed ocular myasthenia gravis that presented with myasthenic crisis-associated TTC. A review of 31 previous cases clearly shows that generalized myasthenia gravis patients tend to be more commonly affected by this disease process. Importantly, myasthenic crisis-associated TTC is linked with significantly higher mortality than myasthenic crisis or TTC alone. Therefore, it is essential that clinicians suspecting myasthenic crisis have a low threshold of suspicion for TTC to promote early recognition and treatment.
